# New quantitative radiographic parameters for vertical and horizontal instability in acromioclavicular joint dislocations

**DOI:** 10.1007/s00167-017-4579-6

**Published:** 2017-05-25

**Authors:** Matthias A. Zumstein, Philippe Schiessl, Benedikt Ambuehl, Lilianna Bolliger, Johannes Weihs, Martin H. Maurer, Beat K. Moor, Michael Schaer, Sumit Raniga

**Affiliations:** 1 Shoulder, Elbow and Orthopaedic Sports Medicine, Department of Orthopaedic Surgery and Traumatology, Inselspital, Bern University Hospital, University of Bern, Freiburgstrasse 18, 3010 Bern, Switzerland; 2 Department of Radiology, Inselspital, Bern University HospitalUniversity of Bern, Freiburgstrasse 18, 3010 Bern, Switzerland

**Keywords:** Acromioclavicular joint, AC joint, AC joint separation, Dislocation, Radiographic parameters, AC–DC, GC–PC, Instability, Horizontal instability, Vertical instability, Intra- and interobserver reliability, Validity, Rockwood classification

## Abstract

**Purpose:**

The aim of this study was to identify the most accurate and reliable quantitative radiographic parameters for assessing vertical and horizontal instability in different Rockwood grades of acromioclavicular joint (ACJ) separations. Furthermore, the effect of projectional variation on these parameters was investigated in obtaining lateral Alexander view radiographs.

**Methods:**

A Sawbone model of a scapula with clavicle was mounted on a holding device, and acromioclavicular dislocations as per the Rockwood classification system were simulated with the addition of horizontal posterior displacement. Projectional variations for each injury type were performed by tilting/rotating the Sawbone construct in the coronal, sagittal or axial plane. Radiographic imaging in the form of an anterior–posterior Zanca view and a lateral Alexander view were taken for each injury type and each projectional variation. Five newly defined radiographic parameters for assessing horizontal and vertical displacement as well as commonly used coracoclavicular distance view were measured. Reliability, validity and the effect of projectional variation were investigated for these radiographic measurements.

**Results:**

All radiographic parameters showed excellent intra- and interobserver reliability. The validity was excellent for the acromial centre line to dorsal clavicle (AC–DC) in vertical displacement and for the glenoid centre line to posterior clavicle (GC–PC) in horizontal displacement, whilst the remaining measurements showed moderate validity. For AC–DC and GC–PC, convergent validity expressed strong correlation to the effective distance and discriminant validity demonstrated its ability to differentiate between various grades of ACJ dislocations. The effect of projectional variation increased with the degree of deviation and was maximal (3 mm) for AC–DC in 20° anteverted malpositioning and for GC–PC in 20° retroverted malpositioning.

**Conclusions:**

AC–DC and the GC–PC are two novel quantitative radiographic parameters of vertical and horizontal instability in ACJ dislocations that demonstrate excellent reliability and validity with reasonable inertness to malpositioning. The use of AC–DC for assessing vertical displacement and GC–PC for assessing horizontal displacement in a single Alexander view is recommended to guide the appropriate management of ACJ dislocations. A better appreciation of the degree of horizontal instability, especially in lower Rockwood grades (II, III) of ACJ dislocations, may improve management of these controversial injuries.

## Introduction

Acromioclavicular joint (ACJ) dislocations are a common injury in the young active population [1–3]. They are invariably classified as per the Rockwood classification [4]. This system is based on the comparative examination of bilateral anterior–posterior stress radiographs and evaluation of the coracoclavicular (CC) distance relative to the uninjured side. This allows an assessment of vertical instability. Several studies have shown that dynamic instability in the horizontal plane is associated with inferior clinical outcomes [5–7]. Except for Type-4 injuries, the Rockwood classification system does not assess horizontal instability. Furthermore, we do not understand what effect horizontal instability may have on the quantitative assessment of vertical instability. The combined posterior–superior dislocation of the clavicle with respect to the acromion may lead to an unpredictable effect on the radiographic CC distance due to projection—a possible underestimation of the CC distance for example, may inappropriately influence non-operative management of potential high grade injuries.

To our knowledge, there is no reliable way of quantifying both vertical and horizontal instability in ACJ dislocations. The majority of radiographic parameters assessing horizontal instability that have been described in the literature to date are semi quantitative and do not account for the projectional differences that may lead to inaccuracies [8–10]. Several authors have proposed the use of different radiographic views to assess horizontal instability [1, 11, 12]. The Alexander view is one such view [13], but the radiographic projection of the ACJ in these views are very sensitive to subtle differences in anatomical position of the ACJ in the coronal, sagittal and axial planes relative to the radiograph beam. This makes comparative assessment (injured side vs uninjured side) with the use of bilateral Alexander views unreliable.

The aim of this study was to identify novel quantitative radiographic parameters for assessing not only vertical but horizontal instability in different Rockwood grades of ACJ dislocations and to investigate the effect of the projectional variation of these parameters in obtaining Alexander view radiographs. In detail, the study includes (1) the analysis of the reliability for the radiographic parameters, (2) the assessment of the validity compared to effective distance and within injury types and (3) the calculation of deviation within projectional variations.

## Materials and methods

### Sawbone modelling of ACJ dislocations

**Fig. 1:**
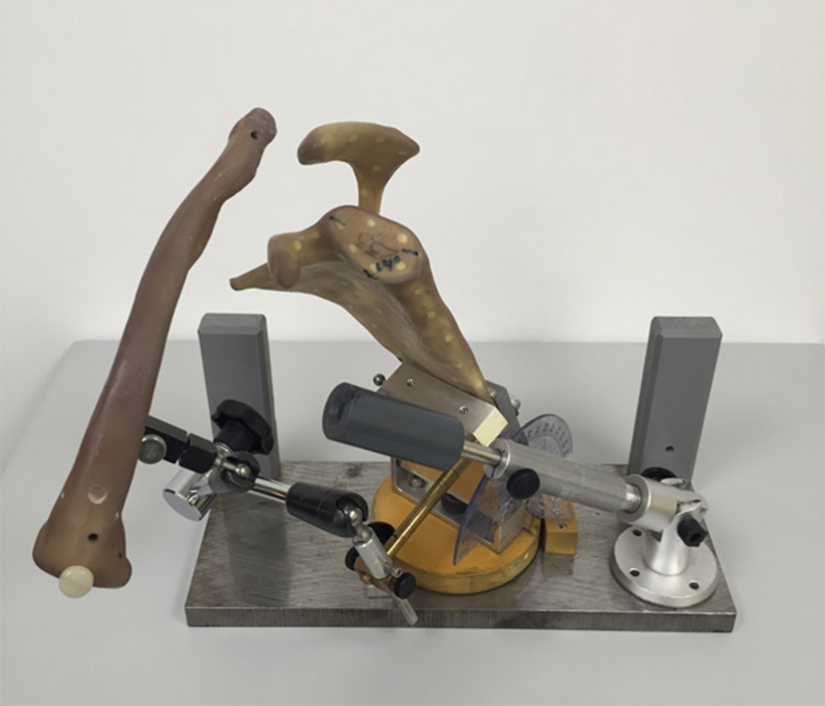
Sawbone modelling. Specially designed holding device where a Sawbone Model was mounted. This model allows to simulate different ACJ dislocation as well as navigation in axial, sagittal and coronal direction according to measurement protocol

A Sawbone model of a scapula with clavicle (Pacific Research Laboratories, Inc., Vashon, WA, USA) was mounted on a specifically designed holding device (Fig. 1). This system allowed precise positioning of the clavicle relative to the acromion in order to simulate ACJ dislocations with both vertical and horizontal displacement. A radiopaque ball was fixed to the lateral border of the acromion along the line of the inferior border of the acromion and the lateral clavicle to allow calibration during radiographic analysis.

The Sawbone model was then setup to simulate ACJ dislocations as per the Rockwood classification system (Grades I to V) with the addition of horizontal posterior displacement for each injury type (Table 1). The degree of displacement simulated for each type of injury was based on the measurement of the height/width of the clavicle. This measurement was then utilized to simulate the appropriate vertical and horizontal instability for each injury type.

**Table 1:** Overview of the seven simulated ACJ dislocations

Simulated ACJ dislocations according to Rockwood classification	Vertical displacement to superior (%)	Horizontal displacement to posterior (%)
Control	0	0
RW II-0	50	0
RW II-25	50	25
RW III-0	100	0
RW III-50	100	50
RW IV-100	200	100
RW V-200	200	200

Rockwood classification (RW) with vertical displacement served as basis (first column) and was combined with the addition of horizontal displacement for each injury type. The degree of displacements are given in percentage based on the measurement of the height/width of the clavicle (= 100%)

### Projectional variations

Projectional variation was simulated for every Rockwood grade of ACJ dislocation. Therefore, the Sawbone construct was tilted in the coronal (medial–lateral) and sagittal (anterior–posterior) planes, and rotated in the axial plane (anteversion-retroversion). The projectional variations for each injury type were simulated with 10° and 20° in any direction, resulting in one neutral position and twelve variations as follows:

- Sagittal—Flexion and Extension—+20°/+ 10°/−10°/−20°
- Coronal—Abduction and Adduction—+20°/+ 10°/−10°/−20°
- Axial—Ante- and Retroversion—+20°/+ 10°/−10°/−20°

### Radiographic imaging

**Fig. 2:**
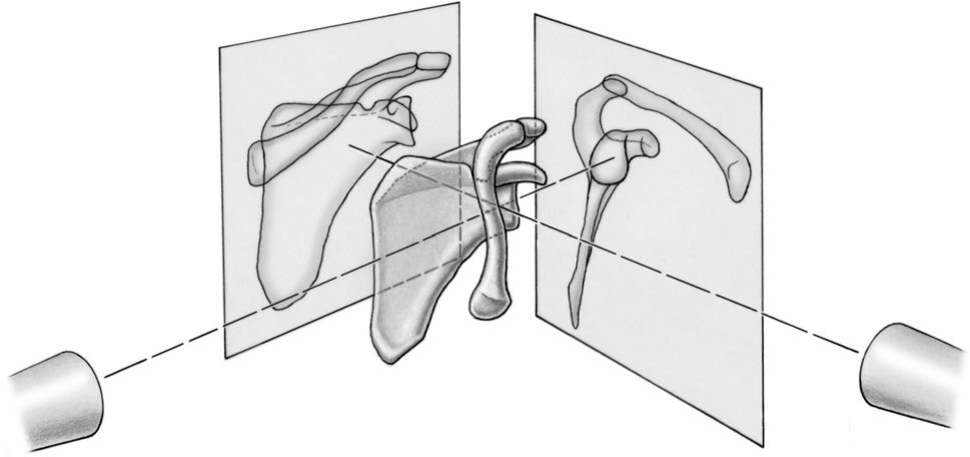
Overview of the radiographic imaging. First, an anterior–posterior Zanca view radiograph was taken with the standardized technique and second, a lateral Alexander view radiograph with an angle of 10° cranial tilt of the beam

Radiographic imaging included computed tomography (CT) scanning and conventional radiography. Once the Sawbone model was setup to simulate a particular Rockwood grade of ACJ dislocation, CT scans were performed on a Siemens Somatom Definition Flash System (Siemens, Erlangen, Germany) with the following scanning parameters: tube voltage 140 kV/; tube current 350mAs; matrix 512 × 512; slice thickness 0.6 mm. Then, a series of radiographs were taken for each injury type with the neutral position and with the simulated projectional variations (Fig. 2). First, an anterior–posterior (AP) Zanca view radiograph [14] was taken with the standardized technique. The radiographic tube to image distance was 120 cm. The central beam was directed to the midpoint of the centre of the glenoid including an angle of 10° cranial tilt of the beam. Second, an lateral Alexander view radiograph [13] focusing on the centre of the glenoid was taken. In total, 2 × 91 radiographs and 7 CT were obtained for further analysis. All images were assigned an identification number in random order and stored in the picture archiving and communications system (PACS).

### Radiographic measurements

**Fig. 3:**
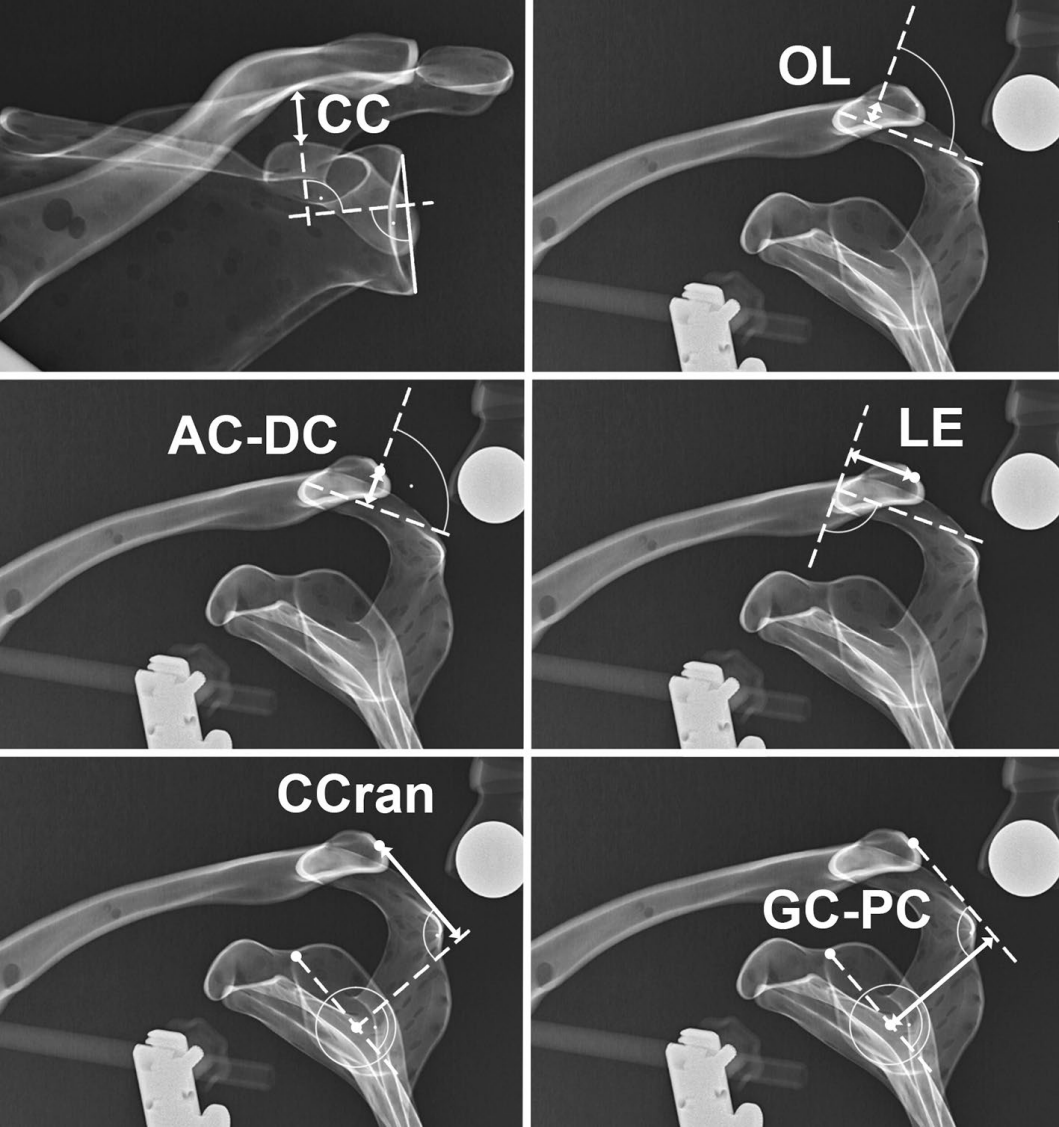
Illustration of the six performed radiographic measurements. On the anterior–posterior Zanca view, coracoclavicular distance (CC) was measured for vertical displacement. On the Alexander view, acromial* centre line* to dorsal clavicle (AC–DC) and centre cranialization (CCran) were measured for vertical displacement and Maximal Overlap (OL), Lateral Extension (LE) and glenoid* centre line* to posterior clavicle (GC–PC) for horizontal displacement

On the AP Zanca view, the commonly used and often published CC distance was measured as the distance between the knee of the coracoid and the inferior cortex of the clavicle for vertical displacement. Five new parameters were defined for radiographic analysis in the lateral Alexander view as shown in Fig. 3. The acromial centre line to dorsal clavicle (AC–DC) and centre cranialization (CCran) for vertical displacement, and maximal overlap (OL) [15], lateral extension (LE) and the glenoid centre line to posterior clavicle (GC–PC) for horizontal displacement.

The AC–DC was defined as the vertical distance between the mid-acromion level and the midpoint of the lateral clavicle, measured perpendicular to the mid-acromion level. The CCran was defined as vertical distance from the centre of the glenoid circle to the midpoint of the lateral clavicle. A line drawn from the centre of the glenoid circle intersecting through the 12 o’clock position on the glenoid face (posterior base of coracoid) was used as the reference. A line perpendicular to this reference line was then drawn from the centre of the glenoid circle. A parallel line to reference line was drawn from the midpoint of the lateral clavicle. The vertical distance from the midpoint of the lateral clavicle with respect to the perpendicular line from the centre of the glenoid circle was then measured as the vertical displacement.

The GC–PC was analogously measured for posterior displacement. The posterior distance on the perpendicular line from the centre of the glenoid to the parallel line through the midpoint of the lateral clavicle was the posterior displacement of the clavicle with respect to the centre of the glenoid. The OL represents the maximal overlap distance of the lateral clavicle and the acromion, and the LE describes the posterior distance between a perpendicular line to the mid-acromion level and the midpoint of the lateral clavicle. All six radiographic parameters were presented in mm and measured in the PACS with an accuracy of one decimal.

### Intra- and interobserver reliability

Intra- and interobserver reliability was assessed using the following radiographs: the neutral rotation radiograph of each grade of ACJ dislocation and additional 4 randomly selected radiographs with 10° tilt/rotation in any direction, resulting in total 35 radiographs. The measurements were taken by three examiners (PS, JW, SR). Each examiner was blinded and independent to the other measurements. One examiner (SR) assessed the measurements two times at an interval of two weeks in order to determine intraobserver reliability.

### Validity

Validity was assessed with determination of convergent validity and discriminant validity. Convergent validity occurs when the scales of a measurement correlate as expected with the related scales of another measurement. For this purpose, the radiographic parameters were compared with the effective distances measured on 3-dimensional reconstructions of CT scans for each grade of dislocation. An independent radiologist who was not involved in the study determined the horizontal and vertical distance between the acromion and clavicle. Discriminant validity is the ability to detect relevant differences between different subgroups and is assessed by calculating the effect size between the different injury types.

The study was done in accordance with the Guidelines of Good Clinical Practice. According to the *Swiss Human Research Act*, this study does not require any IRB approval because neither human beings nor biological material were involved.

### Statistical analysis

Results are expressed as a mean with standard deviation (SD) and with 95% confidence interval (95% CI) where appropriate. Inter- and intraobserver reliability was determined by using the two-way random intraclass correlation coefficient (ICC) assuming single measurement and absolute agreement for each radiographic parameter (ICC (2,1)) [16] and presented with a 95% CI. The ICC values were interpreted with use of the classification suggested by Munro [17] with >0.9 indicates very high reliability. For further analysis, the mean of all observers were used. Convergent validity, indicating whether radiographic parameters correlate to effective distance, was determined by using Pearson correlation coefficient *r*. Correlation coefficient was calculated three times to estimate how rotational deviations influence the result: First calculation with only the perfect projections included (*n* = 7), second calculation with the additional 10 degrees deviations (*n* = 49) and third calculation considers all projection deviations (*n* = 91). The degree of correlation was defined with less than 0.2 equalled poor, 0.2–0.4 low, 0.4–0.6 moderate, 0.6–0.8 good, and >0.8 excellent. Discriminant validity, indicating whether measurements discriminate between the ACJ dislocation groups, was assessed by an independent ANOVA test and the calculation of the effect size with *η* ^*2*^. The sample size to determine the reliability was based on the optimal design for reliability studies as described by Walter et al. [18] Minimum ICC was set at 0.8 and expected at 0.9. With three measurement replicates in 33 samples, a significance level of 0.05 with a power of 80% was obtained. Statistical significance was set as *p* < 0.05. Statistical data analysis was performed with the use of SPSS (IBM SPSS Statistics for Windows, Version 21.0, Armonk, NY, USA: IBM Corp.) and the use of R: a language and environment for statistical computing (R Core Team, 2016. R Foundation for Statistical Computing, Vienna, Austria).

## Results

### Reliability

All radiographic parameters showed excellent intra- and interobserver reliability (Table 2).

**Table 2:** Reliability and validity results for the six radiographic parameters

	Reliability		Validity		
	Intraobserver R.	Interobserver R.	Convergent V.		Discriminant V.
	*ICC 2,1 (95% CI)* [*n* = 35, 2 measures]	*ICC 2,1 (95% CI)* [n = 35, 3 observer]	*Pearson’s r* [n = 7, neutral]	*Pearson’s r* [n = 49/91, w/variation]	*ES η* ^*2*^ [n = 91]
Vertical displacement					
CC	0.956 (0.712–0.993)	0.985 (0.928–0.997)	0.833*	0.778*/0.706*	0.743
AC–DC	0.993 (0.982–0.997)	0.998 (0.996–1.000)	0.972*	0.960*/0.939*	0.952
CCran	0.827 (-0.440–0.956)	0.964 (0.909–0.984)	0.495	0.460*/0.473*	0.905
Horizontal displacement					
OL	0.805 (0.647–0.897)	0.945 (0.906–0.970)	−0.488	−0.433*/−0.385*	0.587
LE	0.925 (0.855–0.961)	0.987 (0.977–0.993)	0.387	0.383*/0.346*	0.713
GC–PC	0.985 (0.970–0.992)	0.995 (0.991–0.997)	0.968*	0.962*/0.952*	0.964

Reliability is calculated for intra- and interobserver measurement and presented with intraclass Coefficient (ICC 2,1) and 95% Confidence Interval (95% CI). Convergent validity are presented by Pearson’s correlation coefficient (*Pearson’s r*) and are calculated for neutral orientation only (*n* = 7), 10° projectional variation included (*n* = 49) and 20° projectional variation included (*n* = 91). Discriminant validity is represented by effect size (*ES η* ^*2*^)

* Values are significant at level <0.05

### Validity

Whereas the validity was excellent for AC–DC in vertical displacement and for GC–PC in horizontal displacement, the remaining measurements showed moderate validity (Table 2).

The convergent validity, which represents the correlation between a radiographic parameter and the effective distance measured on CT, was 0.972 for AC–DC and 0.968 for GC–PC. Despite radiographic projectional variation of up to 20° due to malpositioning, convergent validity remained excellent for AC–DC and GC–PC with only marginal decrease in the correlation coefficient.

The discriminant validity, which represents a parameters ability to distinguish between different grades of ACJ separations, was excellent for AD-DC (0.952) and for GC–PC (0.964). Although CCran also demonstrated good discriminant validity (0.905), this measurements do not increase or decrease with an increase in vertical displacement between the different grades of ACJ separation as illustrated in Table 3. Hence, it has no utility in clinical practice.

**Table 3:** Radiographic parameters and effective CT distance for each injury type

Parameter	Control mean (SD) [mm]	RW II-0 mean (SD) [mm]	RW II-25 mean (SD) [mm]	RW III-0 mean (SD) [mm]	RW III-50 mean (SD) [mm]	RW IV-100 mean (SD) [mm]	RW V-200 mean (SD) [mm]	*p value*
Vertical displacement								
Effective	1	6	6	11.3	11.5	19.2	25	
CC	12.6 (1.9)	17.2 (2.0)	16.7 (1.6)	20.5 (1.8)	17.1 (2.2)	17.5 (1.9)	23.5 (2.1)	<0.001*
AC–DC	2.3 (1.9)	6.9 (1.9)	9.1 (1.8)	11.4 (2.2)	13.0 (1.4)	17.9 (1.3)	33 (3.7)	<0.001*
CCran	48.9 (2.8)	55.2 (1.3)	52.7 (1.3)	55.9 (2.0)	48.5 (1.6)	46.6 (1.7)	66.6 (3.3)	<0.001*
Horizontal displacement								
Effective	1	1	3	1	5.5	11	11	
OL	10.8 (1.0)	11.2 (2.2)	12.3 (2.3)	9.5 (4.0)	12.5 (1.7)	12.4 (1.9)	−5.2 (12.3)	<0.001*
LE	18.6 (3.7)	11.9 (3.2)	17.1 (3.5)	12.8 (3.2)	22.1 (3.3)	27.6 (2.8)	11.8 (5.2)	<0.001*
GC–PC	32.1 (2.0)	32.2 (1.3)	37.7 (2.1)	37.4 (1.5)	43.9 (1.2)	50.9 (0.9)	55.3 (2.4)	<0.001*

Data are presented with mean and standard deviations (SD) as absolute value measured in millimetre (mm). SD derives from projectional variations. Differences were tested for significance with independent ANOVA analysis and presented with p-value

*Values are significant at level <0.05

**Fig. 4:**
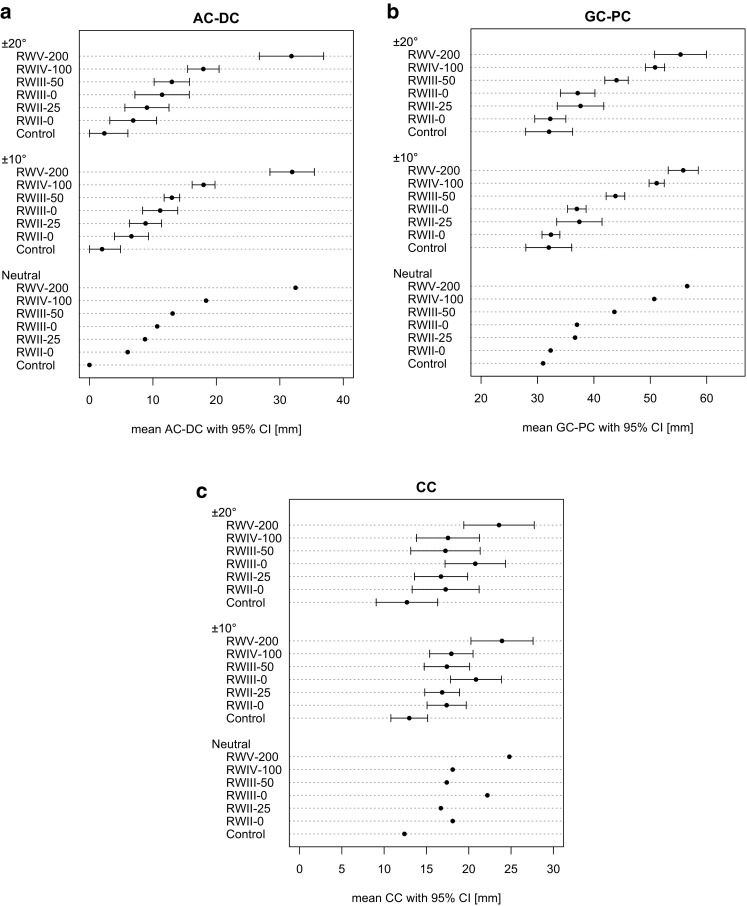
Effect of projectional variation for each type of injury. Mean and 95%CI are presented for each ACJ dislocation group depending of the amount of projectional variations included (neutral *n* = 7, 10 degrees *n* = 49 and 20 degrees *n* = 91). AC–DC is represented in **a** *left*, GC–PC in **b** *middle* and CC in **c** *left*

The individual values with 95% CI for AC–DC (Fig. 4a) and GC–PC (Fig. 4b) demonstrate that these measurements differentiate worsening severity of the ACJ dislocation effectively. In contrast, the CC distance does not differentiate the various grades of injury as well (Fig. 4c). Although the width of the 95% CI for AC–DC and GC–PC increases with the degree of projectional variation, minor deviations are negligible because they are less extensive than differences between injury type. Whereas vertical displacement measured by AC–DC is able to differentiate between all ACJ dislocations groups, the horizontal displacement measured by GC–PC is unable to clearly differentiate between the control and RWII-0 groups, as well as RWII-25 and RWIII-0 groups.

### Projectional variation

**Fig. 5:**
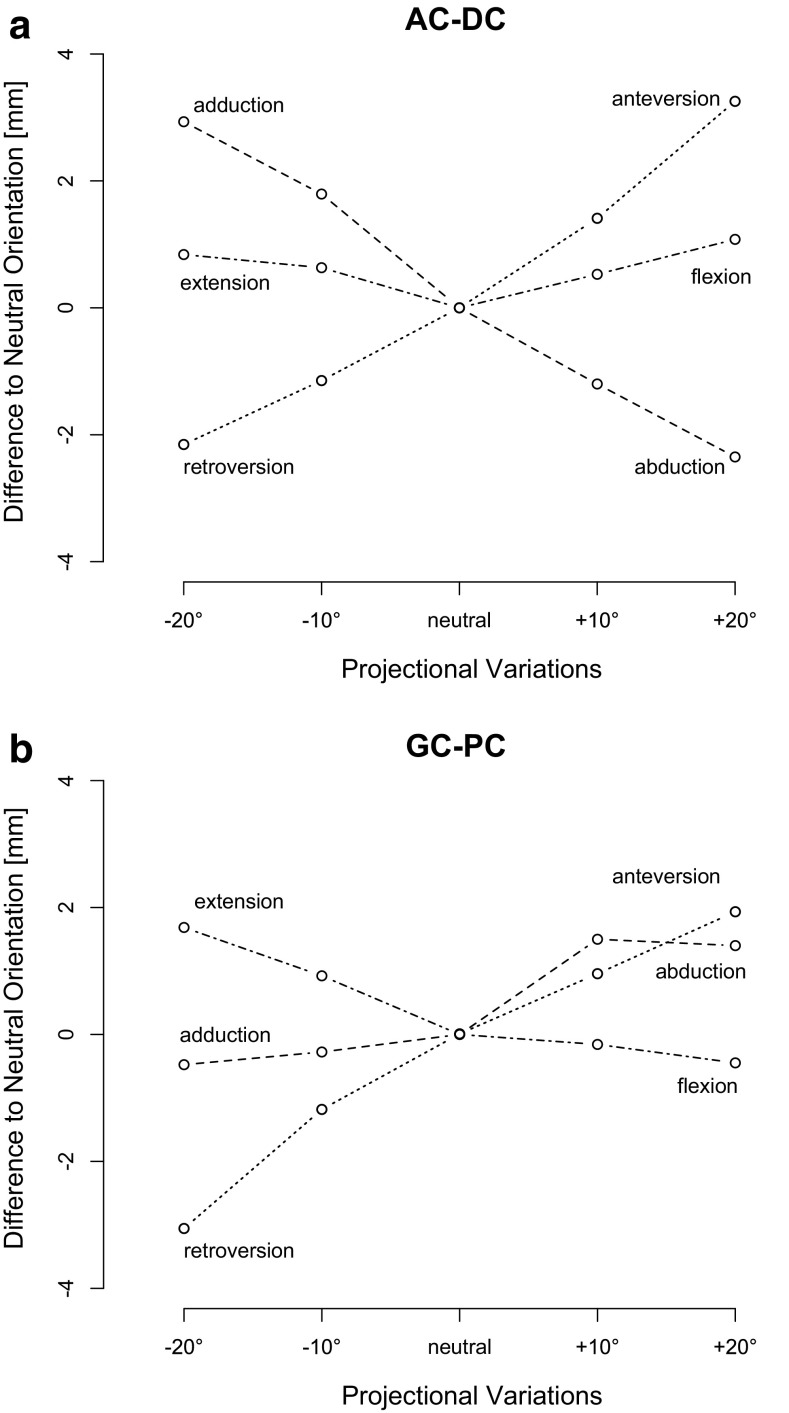
Effect of projectional variation relating to the malposition. Mean deviations of the projectional variations (±10°, ±20°) are shown for AC–DC (**a**) and GC–PC (**b**) compared to neutral. Mean values are calculated including the different injuries. *Lower values* indicate underestimation, *higher values* overestimation. Axial variations are shown in *dotted*, sagittal variations *dot dashed* and coronal variation *dashed*

The effect of projectional variation due to malpositioning error on AC–DC measurements for vertical instability is less than one millimetre for deviation in the sagittal plane, and a maximum of 3.2 mm for 20° of anteversion (Fig. 5a). Malpositioning with abduction and retroversion tends to slightly underestimate the true value, whilst adduction and anteversion leads to a slight overestimation. For GC–PC measurements, a projectional variation of 20° retroversion leads to 3.1 mm difference. (Figure 5b). Extension, anteversion and abduction slightly overestimate the true value to within 2 mm, whilst adduction and flexion demonstrate negligible (< 0.3 mm) deviation.

## Discussion

The most important finding of the present study was the identification of two novel quantitative radiographic parameters of vertical (AC–DC) and (GC–PC) displacement in ACJ dislocations that are reasonably inert to projectional variations and may influence the management of these common injuries.

Despite ACJ separations being frequent injuries, there is still controversy around when surgical intervention is indicated. Although we readily assess vertical instability utilizing the commonly used Rockwood classification, we largely ignore the presence and/or degree of horizontal instability as a factor in managing these injuries either operatively or non-operatively. The assessment of vertical instability in the frontal plane radiographs as per the Rockwood system is known to be affected by projectional variances in obtaining radiographs [4]. Horizontal instability with painful posterior translation of the clavicle may result in further instability and subsequent deterioration in scapulothoracic rhythm [19, 20]. Hence, it has been considered as a potential indication for operative stabilization [21]. The presence and degree of horizontal instability is semi-quantitatively assessed in the lateral Alexander view [7, 22, 23] or by obtaining single or multiple Axillary views [10]. But standard Axillary radiographs have been shown to mimic posterior subluxation of the lateral end of the clavicle [11]. To date, there has been a lack of precise and reliable quantitative methods of assessing ACJ instability that are relatively inert to the projectional variations and that are inherent in obtaining radiographs. The aim of this study was to identify accurate, reliable, quantitative radiographic parameters for the assessment of ACJ separations that are largely unaffected by the inherent projectional variations in obtaining radiographs. Such standardized parameters may significantly influence the management of these common injuries.

Static imaging does not allow us to assess the ligamentous structures that maintain ACJ stability—dynamic or stress views are necessary. Although both Tossy et al. [24] and Rockwood [4] have discussed ACJ separations in the context of the CC and AC ligaments, their radiographic classification systems is largely based on the vertical displacement in the coronal plane, except for the type IV injury. The Rockwood classification, using the CC distances and axillary views have showed a poor inter- and intraobserver reliability and additional 3 dimensional CT reconstruction of the ACJ does not provide further insight [25]. There was poor agreement even among experienced surgeons as confirmed by Kraeutler et al. [26].

This study indicates that the intra- and interobserver agreement on CC distances in the frontal plane for vertical displacement was excellent. However, AC–DC also demonstrated excellent reliability as a quantitative radiographic parameter for vertical instability in the Alexander view with considerably greater validity in comparison with CC distance. Hence, AC–DC was found to have not only the best agreement among surgeons but also the best convergent validity with the strongest correlation between AC–DC measurements and the actual effective vertical displacement. Additionally, the discriminant validity determined by the effect size showed excellent ability of AC–DC in differentiating various grades of ACJ dislocation. In contrast, the CC distance was less discriminant. The measurement of a low CC distance in the Zanca view despite the presence of significant posterior displacement in the horizontal plane suggests that the Rockwood system may underestimate the severity of ACJ separations and therefore misguide surgeons towards non-operative management and poor outcomes [27, 28]. This hypothesis is supported by both, Nemec et al. [29] and Schaefer et al. [30] who demonstrated a different degree of AC ligament injury on MRI in comparison with the original radiographic Rockwood classification system. Interestingly, up to 25% additional ligament injuries were detected by MRI.

The importance of AC ligaments conferring stability was first described in 1917 [31]. Recent studies [19, 20] reemphasized its biomechanical role [19, 20, 32] in maintaining horizontal stability [33, 34]. The lack of attention to horizontal instability may be a reason why non-operative management of even Rockwood type I and II injuries results in clinical failure in up to 27%, which then requires delayed surgical intervention.

This study found that GC–PC had excellent convergent and discriminant validity as a quantitative radiographic parameter for horizontal instability in the lateral Alexander view. And furthermore, it demonstrated excellent intra- and interobserver reliability as well. To our knowledge, the GC–PC is the only quantitative radiographic parameter that allows accurate assessment of the horizontal displacement and is able to differentiate between the various Rockwood injury types. But GC–PC was not able to clearly differentiate between the Control and RWII-0 nor the RWII-25 and RWIII-0. Interestingly, the ISAKOS consensus group [35] has subdivided type III injuries into IIIA (which is the same as RWIII-0 in our study) as well as a type IIIB which describes an overriding clavicle on the cross-body adduction view. Most authors, including the ISAKOS group [7, 36, 37], recommend non-operative management for all these injuries (RWII-0, RWII-25 and RWIII-0) in the general population, hence GC–PC would still make a significant contribution to guiding management decisions despite its limitation in differentiating between the less severe injury types.

Rahm et al. [11], Gastaud et al. [38] and others [10, 39–41] have all shown that despite standardized protocols, classic axillary views are not recommended to assess posterior translation. Our assessment of the effect of projectional variation on our novel quantitative parameters demonstrated that even with 10° and 20° of malpositioning in various planes included, AC–DC and GC–PC continuously increased with increasing severity of ACJ separation. For AC–DC, the maximum mean difference to neutral was 3.2 mm in 20° of anteverted malpositioning, all the other projectional variations lead to a mean difference of less than 2 mm. The various mean projectional variations had even less of an impact on GC–PC with a maximum of 3.1 mm demonstrated with 20° of retroverted malpositioning. A comparative assessment of these quantitative radiographic parameters for ACJ separation to a normal uninjured control side may demonstrate a slight overestimation of AC–DC if the shoulder is too anteverted or adducted. And an underestimation is possible if the shoulder is too abducted or retroverted for AC–DC and anteverted for GC–PC. Both increase in anteversion and retroversion can generally be detected if the glenoid is not centred within the Y formed by the lateral and medial border of the scapula on the Alexander view.

Malpositioning errors may have an impact on the ability to differentiate between RWII and both RWIII injury subtypes in the vertical plane although the ability to differentiate between these less severe injury subtypes may not make a significant impact towards guiding management whilst in the horizontal plane the projectional error has the greatest impact on differentiating between RWII-25 and RWIII0 and both injury types that are treated non-operatively [7, 10, 36, 37, 42–44]. Hence, in terms of clinical decision-making in ACJ separations, projection errors would have the greatest impact on the use of CC distance in the vertical plane.

This study has several limitations. It is an in vitro study which utilizes one Sawbone scapular model in different orientations and therefore it does not fully represent the subtle anatomical variations between patients. A further limitation is the static and not dynamic experimental setup in our study. Absolute distances may change in a cadaver or clinical setup, and our values cannot be directly transferred to clinical practice. The clinical relevance of these new parameters and its role in guiding management of ACJ separations can only be fully understood and validated with a prospective clinical study. Reliable radiographic assessment of vertical and horizontal stability has an important role in guiding management, but the patients’ symptoms, functional demands and clinical examination findings are invaluable. Then, our analysis is based on radiographs with a predefined centre of the radiograph beam. This has become the standard setup for shoulder radiographs and it may not apply to radiographs obtained in other ways. The influence of variations on the radiograph centring and film focus distance was not analysed. This study did, however, analyse the data of different orientations of the model and its influence on these distances which was reported to be minor. This possibility indicates that the variation of the radiograph centring may not have a significant contribution to the overall results. The conclusions are therefore only directly transferable to radiographs centred on the glenoid centre.

## Conclusion

AC–DC and GC–PC are two novel quantitative radiographic parameters of vertical and horizontal instability in ACJ dislocations that demonstrate excellent reliability and validity. They are both measured on lateral Alexander radiographic views with reasonable inertness to projectional errors. The CC distance may underestimate the severity of ACJ separations especially when there is horizontal instability. We recommend the use of AC–DC for assessing vertical displacement and GC–PC for assessing horizontal displacement in a single Alexander view to guide the appropriate management of ACJ separations. A better appreciation of the degree of horizontal instability, especially in lower Rockwood grades (II, III) of ACJ dislocations, may improve management of these controversial injuries.

## List of abbreviations

ACJ: Acromioclavicular Joint
AC: Acromioclavicular
CC: Coracoclavicular
RW: Rockwood Classification
CT: Computed tomography
AP: Anterior–posterior
CC: Distance coracoclavicular distance (radiographic parameter)
AC–DC: Acromial centre line to dorsal clavicle (radiographic parameter)
CCran: Centre cranialization (radiographic parameter)
OL: Maximal Overlap (radiographic parameter)
LE: Lateral Extension (radiographic parameter)
GC–PC: Glenoid centre line to posterior clavicle (radiographic parameter)
ICC: Intraclass coefficient
SD: Standard deviation
95%CI: 95% Confidence Interval
ES: Effect Size
